# Printed Electrochemical Biosensors: Opportunities and Metrological Challenges

**DOI:** 10.3390/bios10110166

**Published:** 2020-11-04

**Authors:** Emilio Sardini, Mauro Serpelloni, Sarah Tonello

**Affiliations:** 1 Department of Information Engineering, University of Brescia, Via Branze 38, 25123 Brescia, Italy; emilio.sardini@unibs.it (E.S.); mauro.serpelloni@unibs.it (M.S.); 2 Department of Information Engineering, University of Padova, Via Gradenigo 6, 35131 Padova, Italy

**Keywords:** printed biosensors, printing technologies, electrochemistry, point-of-care

## Abstract

Printed electrochemical biosensors have recently gained increasing relevance in fields ranging from basic research to home-based point-of-care. Thus, they represent a unique opportunity to enable low-cost, fast, non-invasive and/or continuous monitoring of cells and biomolecules, exploiting their electrical properties. Printing technologies represent powerful tools to combine simpler and more customizable fabrication of biosensors with high resolution, miniaturization and integration with more complex microfluidic and electronics systems. The metrological aspects of those biosensors, such as sensitivity, repeatability and stability, represent very challenging aspects that are required for the assessment of the sensor itself. This review provides an overview of the opportunities of printed electrochemical biosensors in terms of transducing principles, metrological characteristics and the enlargement of the application field. A critical discussion on metrological challenges is then provided, deepening our understanding of the most promising trends in order to overcome them: printed nanostructures to improve the limit of detection, sensitivity and repeatability; printing strategies to improve organic biosensor integration in biological environments; emerging printing methods for non-conventional substrates; microfluidic dispensing to improve repeatability. Finally, an up-to-date analysis of the most recent examples of printed electrochemical biosensors for the main classes of target analytes (live cells, nucleic acids, proteins, metabolites and electrolytes) is reported.

## 1. Introduction

In recent decades, printed electronics, which include all the additive manufacturing techniques to fabricate sensors, circuits, and active and passive electronical components, has gained increasing attention due to advantages in terms of process flexibility, cost and time effectiveness [1,2]. Focusing on the biomedical area, the potential of printed electronics has recently been exploited for the fabrication of bio-sensing electrodes and their conditioning circuits. In this framework, printed electrochemical biosensors have acquired widely recognized relevance in various fields ranging from basic laboratory research to commercially available point-of-care. Thus, the possibility to obtain a sensitive analysis with a time and cost-effective approach, relying on disposable materials and on user-friendly protocols for transduction, is highly demanded by medical personnel, biologists and biotechnologists [3].

Moreover, in basic laboratory research, the possibility given by electrochemical biosensors to correlate electrical quantifiable signals with cell functions or with biomolecule/pathogen concentrations represents an interesting tool for improving the investigation of cellular pathophysiological processes and of their interaction with pathogens [4]. In hospital-based medicine, non-invasive and sensitive bio-sensing gives the possibility to improve the care of patients through ad hoc monitoring during hospitalization, contributing to better detection of bacterial infections [5], and to adjust treatment due to sensitive feedback about patient status [6]. In diagnostics, the possibility to enable the reliable detection of very low concentrations of pathology-related biomarkers, with reduced time and costs with respect to actual biochemical and molecular assays, could bring a revolution in the early diagnosis of pathologies like cancer, cardiac or neurodegenerative diseases [7,8]. Finally, the possibility to integrate those biosensors in standalone platforms (e.g., wearable, point-of-care), usable even by non-experts at home, could provide a powerful contribution to eHealth and telemedicine [9,10,11].

Recent advances in the development of micro- and nanoscale bio-transducers capable of detecting changes down to the molecular level, enabled by technological advances, have strongly accelerated the improvement of the metrological issues still affecting electrochemical biosensors. Those metrological characteristics encompass sensitivity (slope of the calibration plot, given by the ratio between output and input signals), selectivity (ability to correlate changes to a specific analyte, reducing the cross-sensitivity), signal-to-noise ratio (SNR, ratio between the signal of interest and background noise), repeatability (stability of the results among multiple analysis performed under the same conditions) and stability (repeatability in long-term monitoring) [12]. Another relevant useful quantity commonly adopted to compare results in chemistry/biology sensing is the limit of detection (LOD), which express the lowest quantity of an analyte that can be distinguished from the absence of that substance (a blank value) with a stated confidence level (generally 99%). It is estimated from the mean of the blank, the standard deviation (SD) of the blank, the slope (analytical sensitivity) of the calibration plot and a defined confidence factor (usually 3SD) [13,14]. It can also be considered as an indicator of the resolution of the system obtained with a statistical approach, since it is taking into consideration both the contribution of uncertainty and of resolution [13].

Looking at electrochemical biosensors from a metrological perspective, it is undeniable that their characteristics need to be discussed and compared with really competitive counterparts: mass-based and optical biosensors [15,16]. Mass-based devices also referred to as gravimetric biosensors, apply the basic principle of a response to a change in mass, using piezoelectric crystals, in the form of resonating or as surface acoustic wave devices [17]. Their main advantage is their high sensitivity to minimal mass changes, especially for molecules that are neither electroactive nor fluorescent [18,19]. Optical biosensors, both label free and label based, are based on the interaction of optical fields with biorecognition elements, showing well-known levels of sensitivity and specificity [20,21]. Despite those clear advantages and emerging trends in the area of fiber optics [22], both mass-based and optical biosensors show significant challenges in terms of their lack of repeatability, high dependency upon contour variables, high cost, high fragility, limited flexibility, and the portability and integrability of the overall readout system with more complex systems (e.g., point-of-care) [23]. Thus, compared to mass-based [24] and optical [25] biosensors, electrochemical sensors are easier to fabricate and miniaturize, facilitating the possibility of their integration on the same sensing substrate and also customized readout circuits [26]. Regarding metrological performances, despite recent advances in nanostructures, nano-printing strategies and hybrid nano-molecules that have strongly improved the LOD, the main challenges for electrochemical biosensors concern selectivity, repeatability and stability [27]. Recent advances in the area of printing technologies combined with advances in bio- and electrochemistry, nanostructures, solid-state and surface material physics, integrated circuits, microfluidics and data processing offered the possibility to address a whole new generation of electrochemical biosensors [28]; however, these biosensors require attention in relation to their metrological performance.

Compared to the most commonly adopted techniques to fabricate electrochemical biosensors, such as subtractive manufacturing, thin film, vacuum, lithography and electro-based deposition, printing technologies offer unique opportunities in terms of miniaturization, integration in complex systems and ease of customization (Table 1) [29].

**Figure 1:**
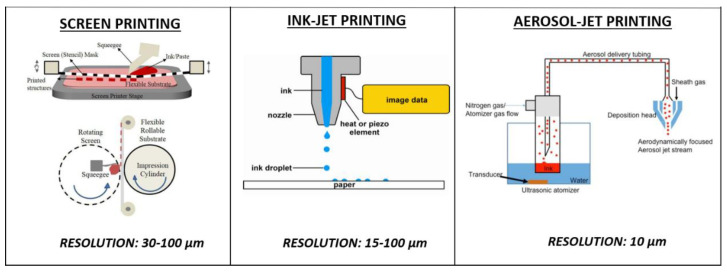
Comparison among fabrication processes to print electrochemical biosensors, in terms of ink dispensing and resolution achieved. Reproduced with permission according to the terms of the Creative Commons Attribution 3.0 license from [43,44,45].

The available equipment for printing technologies ranges from economic devices ensuring very low-cost production, which are ideal for rapid prototyping, to the most expensive ones providing a greater geometrical resolution, which are in some way comparable with standard lithographic methods, but without the need for clean rooms and/or multiple step processes with sacrificial layers [2,25,41]. Overall, the printing technologies employed for fabricating electrochemical sensors can be classified between contact printing (gravure, flexographic, offset, micro-contact dispensing and screen printing (SP)) and non-contact printing (inkjet (IP), aerosol jet printing (AJP), laser-induced forward transfer (LIFT), micro and nano-pen printing). Contact printing encompasses all the mask-based techniques in which patterned structures with inked surfaces and substrate are in physical contact. These techniques ensure high throughput and thus are often (e.g., SP) the most frequently adopted for low-cost and rapidly fabricated biosensors [42]. However, since they are characterized by high material waste, limited resolution and a limited range of materials (substrates, inks and solvents), increasing attention has recently been paid to non-contact printing techniques (also defined as maskless techniques). These technologies are based on ink dispensed through openings or nozzles and define structures by moving the stage in a pre-programmed pattern. Thus, they allow for a reduction in material waste, the simplification of the printing process, an improvement in its control and flexibility and also enable improved resolution, miniaturization and more complex patterns (Figure 1) [43,44].

Along with the advantages discussed, challenges in terms of compatibility among the wide variety of materials used in the fabrication of sensors represent a predominant issue that must be faced to ensure the feasibility and metrological performances of the printed devices. The most recent emerging non-contact techniques [46] are aiming to optimize the processes of ink deposition, reducing the dimensions of droplets (micro- or nano-pen printings [47,48]), through the finest control of printed track width using lasers (LIFT) or by focusing aerosol ink through a stream of gas (AJP) [49]. Additionally, novel sintering methods (e.g., photonic curing) are under investigation to optimize ink post-processing. These emerging techniques are thus trying to face the challenges in terms of conductivity, repeatability and standardization that are still openly affecting printed biosensors when compared with their bulk counterparts [50]. Additionally, the possibility to combine and customize different materials and to exploit novel curing methods with respect to other traditional techniques (e.g., laser cutting, machining) opens the way for the effective integration of biosensing with directly printed microfluidic circuits (e.g., paper based, polymer based) and embedded electronics (insulating layer and conductive tracks), with consequently improved costs and time effectiveness [4,9,51].

Considering this, the aim of this review is to provide an up-to-date picture of the state of the art of printed electrochemical biosensors. First of all, this paper focuses on the opportunities offered by printing technologies for electrochemical biosensors in terms of transducing principles. Following this, a discussion on the main metrological challenges of printed electrochemical biosensors is performed. In particular, we focus on how enhancing the printing approach, combined with the most innovative technologies in terms of nanostructures, microfluidic and non-conventional substrates, is opening up promising avenues through which to face those challenges. Finally, a review of the most upcoming trends of printed biosensors for the main target analytes (cells, nucleic acids, proteins, metabolites and electrolytes) is provided.

## 2. Transducing Principles of Printed Electrochemical Biosensors

The transducing principles of printed electrochemical biosensors can be grouped into three main classes: amperometric, impedimetric and potentiometric [52]. Common advantages brought by printing technologies to all three classes are related to the miniaturization of the electrodes, to the use of nanostructured inks, to printed microfluidic paths and via the extension to non-conventional substrates.

Thanks to printing technologies, both three-electrode (for amperometric) and two-electrode (for impedimetric and potentiometric) conformations, traditionally implemented with solid electrodes in a baker containing several milliliters of samples, can be easily miniaturized onto a small substrate, ensuring a reduction in the required sample volume from milliliters to a variable range within picoliters and microliters [49]. Moreover, the capacitive background current associated with the charging of the double layer is reduced proportionally to the reduction in the surface area of the conductive electrodes. The resistive drop in the electrode–solution system is reduced by shortening the ionic current path in miniaturized cells. Overall, those elements contribute to reducing the interfering noise coupled to the electrodes. The reduced time constant coming from reduced capacitance and resistance enables faster electron transfer kinetics monitoring.

Printing technologies enable an easier fabrication of microfluidic circuits. This possibility, combined with high-resolution nanostructured coatings, enhances the accuracy and sensitivity. In fact, thanks to the high accuracy of sample delivery to the sensing area and to the presence of nanowires and nanospheres, the interaction between the analyte and the electrode active area is enhanced, changing it from a 1D planar diffusion to a more uniform 2D or 3D diffusion. The use of nanoinks allows to increase the surface to volume ratio, increasing the active area useful for redox current detection, for impedance variation or charge accumulation detection, bringing an improvement in terms of overall sensitivity. Furthermore, the highest control obtained in these microsystems in terms of sample dispensing, ink and coating deposition can also improve the repeatability of the electrochemical measurements [53,54]. Overall, the combination of the reduction in the interference noise processes and the enhancement of the transducing effect of the measurand achievable in printed miniaturized integrated biosensors increases the signal-to-noise ratio of such bioanalytical systems [55,56].

In addition to working electrodes (WE), the potential of printing techniques also needs to be exploited for improving counter (CE) and reference electrodes (RE), which require particular attention when aiming for electrochemical cell miniaturization [57]. CE represents the element required to complete the circuit with the WE, thus allowing the charge coming from the reaction on WE to flow and be read [58]. Consequently, its size should be much larger than the WE to ensure no current limitations arise. Thus, nanostructures and complex geometries made available by emerging printing are under investigation to increase the surface to volume ratio and to guarantee proper control of the electrical parameters of the cell during the analysis [59]. Regarding RE, it is the element that needs to be kept at a constant potential during all the analyses, to control the potential of WE (e.g., in voltammetry) or to allow measurement of an indicator electrode (e.g., in potentiometry). Thus, attention is being paid to novel materials and curing strategies to improve the stability of RE and limit the influence of surrounding conditions [60].

Despite these common advantages, due to significative differences in terms of speed, sensitivity and selectivity among amperometric, impedimetric and potentiometric biosensors, the specific potential offered by printing technologies for each class needs to be discussed, considering their intrinsic characteristics (Table 2) [15].

Next, the basic working principles, advantages and disadvantages of amperometric, impedimetric and potentiometric biosensors will be overviewed, focusing on the specific contribution and improvement brought by the printing approach to each method. For more extensive and theoretical details of each electrochemical technique, out of the scope of this review, we suggest that the reader deepens their knowledge of this theoretical topic in the related literature [61].

### 2.1. Amperometric

In amperometry, a three-electrode conformation is used, comprising a WE, CE and RE electrode. WE potential is controlled through a signal from a generator and the current resulting from the oxidation and/or the reduction reaction of electroactive molecules exchanging electrons with the WE conductive surface are then measured in the loop closed by the cell. If the signal coming from the generator is varied, then the methods belong to the sub-class of voltammetry [61].

The main challenges of printed amperometric biosensors still refer to cross-sensitivity, the interferences of the buffer composition and the effect of the surrounding environment [70]. Concerning the influence of contour variables, the most challenging aspects refer to interfering molecules (inks, mediators, labels) with similar potential. Concerning implantable electrodes or analyses performed on biological fluids, a relevant issue is the electrode fouling by non-target proteins and biomolecules, which can limit direct electrode exchange. Furthermore, the accuracy and stability of the currents measured are particularly challenging for both short and long-term measurements [71]. Static measurements, in the absence of stirring and without proper fluidics, can be easily affected by saturation due to species accumulation, by difficult low current detection due to double-layer capacitance or by a decrease in electrode performances due to the degradation of ink or of the ink–substrate bonding [72].

A smart combination of high-resolution nanostructure direct printing with peculiar techniques able to enhance low faradaic currents and not background processes (e.g., differential pulse voltammetry) can help to face those issues, reaching LOD < 10–12 M, the lowest among electrochemical techniques [52]. Finally, focusing on biosensor selectivity, cross-sensitivity of different species can be improved thanks to the flexibility in ink preparation. The possibility to directly print selective electroactive labels allows to enhance the selectivity of currents resulting from voltammetries using nanoparticles and nanostructures as electroactive labels (limiting the need for additional markers) [73] and to improve repeatability due to better control of the deposition process.

### 2.2. Impedimetric

Impedimetric biosensors are based on the direct correlation of impedance changes with changes in terms of target analyte concentration, without requiring additional labels or biomolecule electroactivity. After applying an alternate voltage to the two electrodes (WE and CE), with a constant amplitude (usually between 5 and 10 mV) and a defined frequency range-, the resulting alternate current is measured and the overall impedance (Z) correlated with analyte concentration [69]. They provide the result directly, without requiring the electroactivity of the target analyte. Impedimetric biosensors based on the principle that biomolecules bound onto a printed conductive surface are acting as insulators (e.g., adherent cells proliferation monitoring) fall in the subclass of reactive [74]; the ones based on the measurement of electrolytic conductivity to monitor the progress of a chemical ionic reaction instead fall in the class of conductometric [68].

Among the most important advantages of impedimetric biosensors compared to other classes are the low voltages employed, which do not damage or disturb most bio-recognition layers [75]. From the point of view of the target analyte, the small excitation signals adopted cause small amplitude perturbations from the steady state, which makes this method optimal to monitor in real time the dynamics of biomolecule interactions and the pathophysiological processes of living cells, without significant alterations to the ionic balance in the extracellular space [53,54,76]. Furthermore, from the point of view of materials, this low invasiveness gives the possibility to explore novel non-conventional organic conductive materials (e.g., conductive functionalized polymers or small molecule organic semiconductors) with peculiar surface modifications that can enhance the sensitivity and the LOD of the analysis [77].

The challenging aspects of impedimetric biosensors are the strong influence of pH, temperature, buffer characteristics or non-reacting ions on measurement accuracy and repeatability [76,78], the worse detection limits compared to potentiometric or amperometric methods (usually around 10^−8^ M) and the sources of error due to double-layer capacitance and electrode polarization [68]. Furthermore, the wide spectrum of frequencies of the applied voltage implies a very small power at each frequency and, consequently, a limited SNR of the impedance measurement with respect to other electrochemical techniques [79].

The opportunities of the printing approach for impedimetric biosensing mainly refer to the possibility to exploit novel nanostructured inks to enhance SNR and to the availability of biocompatible organic inks to improve the integration of sensing elements in biological environments. Thus, due to the limited invasiveness of the technique, printable organic and degradable inks can also be deposited on the electrode to investigate live cells, allowing impedimetric monitoring during a long-term culture both in 2D and 3D environments [80,81].

### 2.3. Potentiometric

In potentiometric biosensors, the measurement is performed in zero-current conditions, with a two-electrode structure, without the need for a generator or current measurement device. The voltage across WE and RE is measured with a high-input impedance device, to minimize the contribution of the ohmic potential drop to the total difference in potential. The potential of WE, thanks to an accumulation of charged molecules (ions), exclusively depends on the analytical concentration of the analyte in the gas or solution phase, while the RE is needed to provide a defined reference potential [62].

Those biosensors can be easily miniaturized and integrated in all printed devices since they require low-cost measurement instrumentation. Due to the simple electronic conditioning circuit, potentiometric biosensors show a rapid response, ease of use and robustness. On the contrary, their main intrinsic challenges are related to their non-specificity, to the influence on temperature variation, to the need for frequent re-calibration and to false positives due to interfering charged molecules in solution [60,61,82].

Thanks to the progress of additive manufacturing, printed potentiometric biosensors are undergoing a renaissance, with improvements in the detection limits (down to ~10^−8^ M) and selectivity enabled by the introduction of novel materials and the integrability of these sensing concepts with wearable and implantable devices [67]. The possibility to fabricate miniaturized electrodes with customized inks could provide improvements in terms of the stability of RE, tuning the ink composition [57,60] and the selectivity of the approach, directly printing selective coatings to substitute for the selective membranes that are traditionally adopted [83]. Other great opportunities provided by potentiometric measurements combined with printing technologies refer to the possibility to realize innovative sensors on degradable or biological substrates (directly on the skin or implanted in the human body) due to the sensing principle at zero current, which limits the possible perturbation in the sensing area [84].

## 3. Discussion of Opportunities of Printing Technologies and Metrological Challenges of Electrochemical Biosensing

The metrological characteristics of electrochemical biosensors represent the main challenges still slowing down their maturity for robust comparisons within different scientific experimental results and for final reliable use in clinical settings, laboratories and point-of-care applications. Thus, the high sensitivity, low uncertainty, high repeatability, low cross-sensitivity of environmental influence and long-term stability are all essential requirements to performing a meaningful comparison between different repetitions of the same experiments not only at different times, but also within different laboratories [49,85]. Considering the framework described, printed electrochemical biosensors still present large areas for improvement in terms of both metrological performances and application conditions. In these terms, the main opportunities of the printing approach are discussed as powerful tools to improve the metrological performances of biosensors not only in terms of LOD, sensitivity, selectivity, repeatability and stability, but also to enlarge their field of application in environments with non-optimal working conditions (e.g., high humidity, salinity or biologically degradable environments).

### 3.1. Printed Nanostructures to Improve LOD, Sensitivity and Repeatability

**Figure 2:**
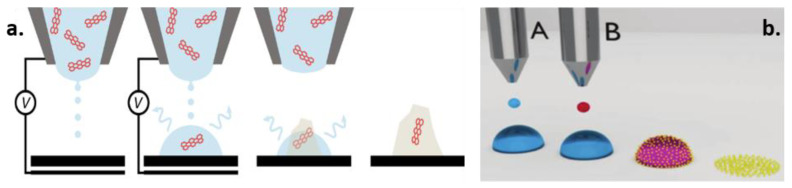
Two examples of strategies to enable finest control of nanostructure printing: (**a**) a schematic of the main steps of on-demand electrohydrodynamic dropwise deposition, solvent evaporation and crystallization, capturing a single molecule in the crystallized deposit and thus achieving oriented nano-molecules [89]; (**b**) how two-step printing strategies with supporting printable materials can help to enhance the uniformity of printed nanostructures [90]. Figures reproduced with copyright permission from John Wiley and Sons [89,90].

Printable inks offer interesting opportunities for customization due to the wide variety of nanostructures and biomolecules that can be incorporated. As highlighted by recent research [86,87], even with the same chemical composition, electrode superficial nano-structuration strongly influences the properties of the finally fabricated biosensors, in terms of both LOD and sensitivity, due to the increase in the active area available for interaction with nano-molecules [88]. Additionally, the possibility of achieving a uniform distribution of nanostructures through the use of multiple supporting printable materials, and of improving the orientation of nano-molecules (e.g., DNA, RNA, antibodies, aptamers) thanks to nano-printing methods [89,90], can have relevant impacts on improving the effectiveness of nanostructure–biomolecule interactions, with the consequent enhancement of the sensitivity, repeatability and LOD of the measurement (Examples in Figure 2).

The use of printed nanostructures was demonstrated to improve the quantifiable LOD, from µM levels commonly observed with bulk electrodes down to nM or even pM levels (lower than traditional gold standard techniques such as ELISA) [46,51]. LOD improvement was shown to be strongly dependent on the type, size and composition of the nanostructures, and to be enhanced when relying on a combination of different nanostructured materials [91]. Furthermore, most of the leading research in electrode nano-structuring has recently confirmed that an accurate micrometric control of nanostructure deposition onto electrodes through micro and nano-printing strategies also represents a winning strategy to lower the relative standard deviation of the overall measurement (<5.0 % compared with the common relative standard deviation (RSD) of 20% registered in electrochemical sensing without control of surface material deposition) [46]. Increasing attention has recently been addressed to the investigation of novel materials and shapes that improves the metrological aspects of LOD, SNR and sensitivity. The use of nano-cubes of novel graphene-based nanostructures, realized with a combination of different materials [92], to enhance cell–biosensor interaction [93], and of printed nanostructures combined with novel curing techniques, have been highlighted in the recent literature as promising to improve the performance of paper-based biosensors [94]. Furthermore, in [95,96], specific comparisons in terms of sensitivity were performed among carbon nanotubes, as well as fullerene and platinum printed nanostructured electrochemical sensors, demonstrating the combined effect of the chemistry, shape, dimension and deposition techniques of the nanostructures on LOD and repeatability. An improvement in the LOD in quantifying IL-8 (from 2 ng/mL to 0.38 ng/mL) and p53 proteins (from 2 ug/mL to 100 ng/mL) could be obtained with nanostructured biosensors with respect to their non-nanostructured counterparts. Interestingly, in [97,98], carbon nanotubes and other functional nanomaterials were shown as also being useful to improve the SNR of electrochemical techniques, since they can, on the one hand, provide excellent electrical conductivity and promote radial diffusion and, on the other, reduce the area of double-layer capacitances. Finally, in [99], comparing different nanostructure deposition strategies while quantifying the very same protein with the same protocol, direct nanostructure printing through AJP deposition was demonstrated as the most sensitive and reproducible technique. It was thus demonstrated that a higher spatial accuracy in the deposition of nanostructures brings improvements both in terms of LOD (improved from (LOD from 2.1 to 0.3 ng/mL) and in the relative standard deviation (RSD, reduced from 50% to 10%), with promising results possibly extended to electrochemical sensors for several diagnostic and medical applications.

### 3.2. Printing Strategies to Improve Organic Biosensors Integration in Biological Environments

One of the areas that has recently gained much attention is the use of biosensors directly embedded in biological environments (implanted in the human body or integrated in cell culture) to obtain reliable feedback from biosensors. In addition to printability and biocompatibility, essential requirements in these applications relate to the adaptability of biosensor elements (e.g., inks, coatings or conditioning circuits) to an environment traditionally harsh for electrical instrumentation, with high humidity and salinity at physiological temperature (around 37 °C).

In this framework, despite the fact that inorganic materials would be commonly preferred due to their higher stability and metrological performances, in the recent literature, growing interest has been addressed to the use of organic printable materials due to their higher biocompatibility and non-invasiveness. In particular, conductive polymers [100], carbonaceous materials [101] and organic semiconductors (poly(3,4-ethylenedioxythiophene) polystyrene sulfonate (PEDOT:PSS), Triisopropylsilyl ether (TIPS)) [102,103,104] all represent attractive candidates due to their low cost, good compatibility with most of the printing process and customizable chemical composition [105].

Despite the fact that several examples have been proposed in areas ranging from cell monitoring to implantable devices [105,106], the main metrological challenges refer to repeatability (often higher than 10%), SNR (often lower than 20), due to intrinsic variations in the background impedance, and the stability of the electronic performance over long periods (most of the works demonstrated only a few days, while feedback on cell cultures would be interesting over longer periods of a few weeks) [97,107]. Only facing those metrological challenges can ensure the intra- and inter-laboratory repeatability required for biosensor validation, opening the way to a whole new world of biosensing that is more biomimetic and integrated with living environments [108].

To this end, specific attention has recently been addressed to exploiting the potential of printing technologies for customizing ink preparation, deposition and curing for both electrodes and coatings [33].

Regarding ink customization, the possibility to tune the ink chemical composition of conductive polymers (e.g., PEDOT:PSS, polyaniline, TIPS-pentacene) represents a powerful strategy that could lead to controlling the metrological performance of biosensors through a finer control of electrode material solubility and degradability, in agreement with target analyte dynamics [109]. Promising examples have demonstrated conductive inks for embedding sensing elements into the human body or 3D scaffolds [110,111], or investigating organic semiconductors (TIPS-pentacene) to realize transistors for monitoring neural cell culture activities, due to their combined printability, biocompatibility and degradability [112].

Another opportunity of the printed approach refers to the possibility to directly print customized coatings onto conductive electrodes. Moreover, an improvement in terms of stability can be brought by printing protecting material (e.g., UV-curable polymers or dielectric layers) to avoid direct contact with ions or water. Furthermore, promising results in terms of repeatability were obtained with the micro and nano-printing of enzymes [113], proteins [114] or cells into scaffolds [115].

**Figure 3:**
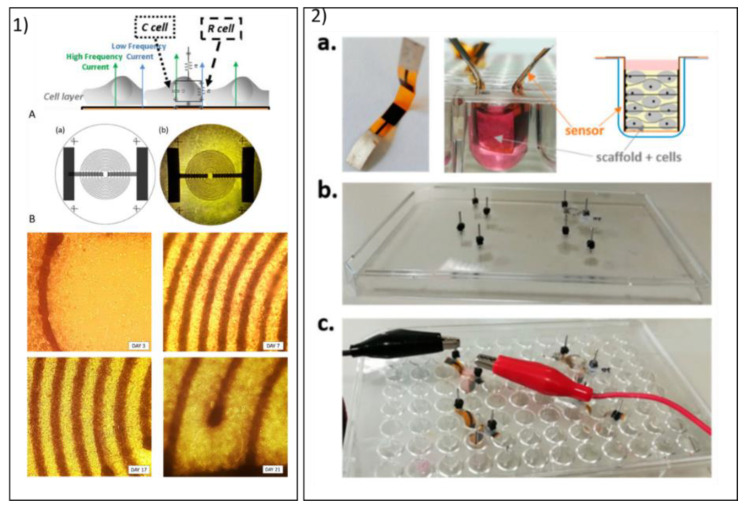
Example of how aerosol jet printing (AJP) biosensors fabricated with organic carbon-based ink designed to enable long-term noninvasive monitoring of cell cultures: (**1**) example of interdigitated carbon-based electrodes customized for multi-well plates for 2D monitoring of the differentiation of CACO-2 cells [116]. Reproduced with copyright permission from Elsevier. (**2**) The set up proposed to monitor mesenchymal stromal cells through foldable parallel carbon electrodes directly within 3D scaffolds [80]. Reproduced from an open access publication.

Regarding stability and SNR during long-term cell monitoring, increasing attention has also been recently addressed to nanostructures and to emerging ink deposition and curing (e.g., AJP) to improve the stability of an effective ink–substrate interaction and consequently of the metrological performance [80,116]. A promising strategy to enhance SNR demonstrated the introduction of carbonaceous nanostructures and the use of emerging micro- and nano-printing techniques to enable their uniform distribution [117,118]. Interesting results from [97,119] showed a five-fold improvement in the standard SNR. Among the emerging technologies, AJP was shown to be effective in fabricating printed carbon electrode that were integrable with glassware as modular systems to monitor the growth and differentiation of human colorectal adenocarcinoma cells (CACO-2) in static 2D cultures [116], and the proliferation of mesenchymal stem cells into 3D scaffolds [80], with stable performances over 21 days of culture (Figure 3). Furthermore, contactless technologies combined with proper surface treatment were also demonstrated to achieve stability in organic carbon inks during dynamic myocyte 2D cultures using stretchable substrates, with a sensitivity of 80 Ω/cell) and a RSD around 20% [120].

### 3.3. Emerging Printing Technologies for Non-Conventional Substrates

**Figure 4:**
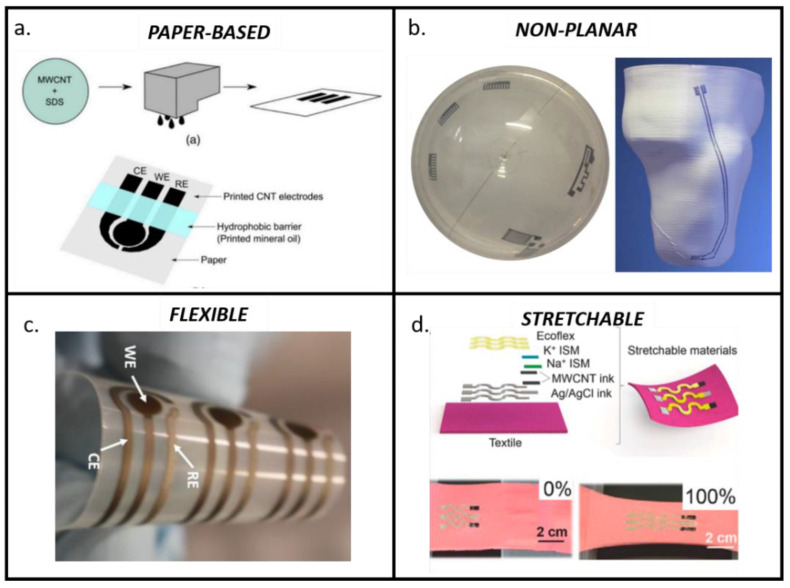
Summary of the main classes of non-conventional substrates enabled by printing technologies: (**a**) paper-based biosensors, often enhanced by nanostructured, as reviewed in [125]; (**b**) biosensors printed on non-planar surfaces, examples presented in [126,127]; (**c**) example of three-electrodes layout for histamine detection printed onto a flexible substrate, [128]; (**d**) a recent example of electrolyte detection for printing electrochemical sensors for wearable applications onto highly stretchable substrates, reproduced by [129]. All figures were adapted from open access papers cited under the Creative Commons license.

Printing technologies allow for the exploitation of a wider variety of substrates compared to traditional techniques. In recent years, several emerging methods have been proposed, enabling greater control of multiple degrees of freedom and also droplet dimension, with a direct effect on resolution with respect to traditional techniques such as SP or IP. These techniques (e.g., micro and nano dispensing, AJP) allowed researchers to enlarge the substrates available from traditional rigid and planar substrates (e.g., ceramic or silica) to non-conventional substrates (e.g., plastic, paper or stretchable substrates). Among non-conventional substrates, paper-based substrates, stretchable and 3D substrates represent the most investigated ones to enable the integration of sensing elements into disposable devices, into substrates undergoing mechanical stretching and on the irregular surfaces of complex structures [121,122] (Figure 4).

Despite clear advantages over rigid traditional substrates, the efficiency of the production and the ease of processing on those non-conventional substrates must be improved before allowing commercialization [123]. Several metrological challenges relate to the performance in terms of the repeatability (with an RSD higher than 10% preventing commercialization [124]) and sensitivity of electrochemical biosensors realized on non-conventional and 3D substrates. In this way, particular attention to the electronic transducing aspects of fully printed devices onto non-conventional substrates have been placed under investigation to correlate the response of fully printed devices with the specific properties of the printed material and on the geometrical characteristics of the electrodes [11].

Novel emerging methods for ink dispensing and curing are trying to face these challenges by improving the performances of biosensors realized on unconventional substrates. In particular, novel, non-contact printing techniques (e.g., AJP, nano dispensing) can achieve resolutions of a few micrometers, along with very good accuracy and repeatability even on materials with poor porosity or with irregular surfaces [46,130]. Regarding the use of stretchable substrates, increasing attention has also been recently addressed to the opportunities and limitations of stretchable inks and substrates [131], highlighting that both geometry optimization and perfect matching between stretchable substrates and inks should be carefully addressed in order to guarantee an optimal performance during all the different phases [132].

**Figure 5:**
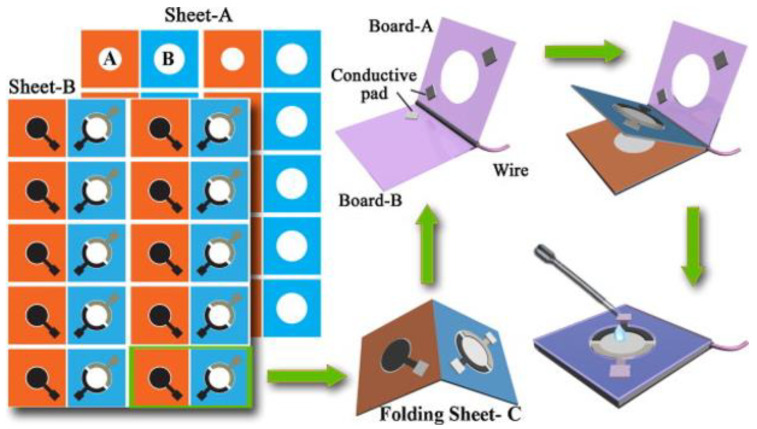
Interesting example of paper-based printed origami biosensors: after multi-plane printing, electrode folding ensures better control of the sample and higher repeatability of the measurement. Reproduced from [143] with copyright permission from Elsevier.

Regarding the disposability and optimization of cost effectiveness, paper represents the most promising substrate to combine cost effectiveness with intrinsic capillary properties in order to improve sample flow control [125,133,134,135]. Furthermore, a paper substrate can truly provide environment friendliness for electrochemical sensors, making them disposable while respecting the standard of the green era and the circular economy [136]. Paper has been used for more than a century in analytical and bioanalytical devices and, nowadays, recent advances in developing paper-based immunosensors, aptasensors and genosensors are highlighted as very promising solutions that combine sensitivity with low cost and disposability [137]. The high performance reached in terms of ink deposition by techniques such as micro-dispensing or AJP is paving the way to the fabrication of low-cost disposable biosensors with metrological accuracy, repeatability and stability comparable with their traditional counterparts [137,138]. Interesting examples are under investigation in order to achieve a combination of biosensing elements [139] and complete circuit fabrication [140] onto cellulose substrates, attracting attention for enabling smart food monitoring into disposable paper-based packaging. Resistivity values of 26.3 × 10^−8^ Ω⋅m on chromatographic paper, 22.3 × 10^−8^ Ω⋅m on photopaper and of 13.1 × 10^−8^ Ω⋅m on cardboard were obtained by AJP. These values are comparable with the range of resistivities obtained with similar inks on conventional substrates (from 4 × 10^−8^ Ω⋅m to 44 × 10^−8^ Ω⋅m depending on deposition and curing parameters) [140,141]. This represent a promising result for integrating electronic tracks on disposable substrates for food packaging, wearables or point-of-care. Furthermore, a combination of paper-based substrates with nanostructures, with origami architectures and with sensitive electrochemical techniques such as Differential Pulse Voltammetry (DPV) or Anodic Stripping Voltammetry (ASV), is enabling researchers to reach a competitive limit of detection in the order of fM [142,143] (Figure 5).

Regarding irregular surfaces, micro and nano-dispensing printing techniques are also a great opportunity for producing biosensors directly onto 3D surfaces [144,145]. These emerging methods, in addition to an optimal control on multiple degrees of freedom, allow for rapid and more effective ink drying, sintering and curing over a wide range of substrates, aspects that are required to improve ink adhesion on irregular surfaces, as well as its conductivity and stability [138]. Thus, differently from flat 2D surfaces, when printing on surfaces with high inclination and rugosity in addition to standard post-printing curing, particular care should also be addressed to primers or to ink drying and/or polymerization during printing, to enable the optimal adhesion of ink on those surfaces [126,127,146]. This provides the possibility to directly integrate repeatable and stable sensors onto already-fabricated products (as in Figure 4b), without the need for attaching external electrodes [99,147,148], but also to integrate highly conductive printed tracks of customized conditioning electronics in an all-in-one structure using a single fabrication technique [149]. This represents an advantage, both for point-of-care applications and for wearable devices, to limit the obtrusiveness during a long-term recording of the patient.

### 3.4. Microfluidic Dispensing to Improve Repeatability

Printing technologies offer unique opportunities in terms of biosensor integration with customized microfluidics, with embedded conditioning electronics or with multisensory platforms. This allows to take a step forward from printed biosensors to standalone printed biosensor platforms, thanks to optimal process flexibility and to the wide range of materials available [150].

An aspect of predominant relevance for achieving accurate biosensing on miniaturized electrodes and for continuous analysis is to ensure proper sample management and control. Three-dimensional printing strategies could create a revolution in this sense, providing the possibility to directly realize both microfluidic systems for sample preparation/distribution and conductive electrodes within the same printing session. This represents a great improvement in terms of lowering circuit fabrication costs and the time for complete platform production. Two main methods to manage and control the sample under analysis are under investigation: (i) the fabrication of support-free microfluidic circuits on the same sensor chip; (ii) the exploitation of the peculiar capillarity of the substrate, such as paper. The first category refers to polymer-based channels that can be fabricated using UV-curable materials. Interesting examples have been shown not only for sample distribution [113], but also in combination with novel nanomaterials to incorporate the filtration, concentration and amplification of the analyte directly within the chip before reaching the measurement point [151]. The second category refers to the fabrication of lateral-flow paper-based assays, in which the paper capillarity is exploited to guarantee the efficient flow of the sample, which is better controlled with customized hydrophilic paths printed with wax or other hydrophobic materials on paper substrates [45,152]. The choice of one with respect to the other category mainly refer to the requirement in terms of the accuracy of fluid control, of the scalability of the device and of the cost of the fabrication.

**Figure 6:**
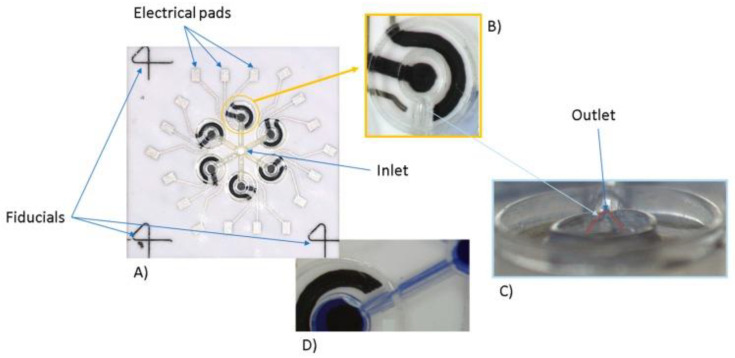
Example of use of fully printed integrated biosensors and microfluidic circuit realized with AJP. The figure represents the platform realized in [113], in which all the elements (electrodes, conductive tracks and polymer-based microfluidic channels) were fabricated and fully printed with the AJP technique. In details: (**A**) Layout of the complete platform; (**B**) Zoom of a single electrochemical cell; (**C**) Detail of microfluidic inlet; (**D**) Example of liquid control in each sensing point. This figure was reproduced from an open access publication [113].

These opportunities, ensuring an efficient and controlled delivery of small sample volumes, could represent a key element to increase repeatability among different batches and laboratories. Furthermore, realizing a proper microfluidic circuit able to distribute equal parts of the sample on multiple sensing areas could help to improve not only the repeatability, but also the sensitivity of the overall analysis. An example of how technologies for printed electronics are playing a relevant role in facing those metrological challenges can be found in [113] (Figure 6). The AJP strategy was applied therein to improve the repeatability and sensitivity of glucose sensing. Through a single printing process, a complete platform was developed, including the microfluidic circuit, the electrodes and enzyme-based electrode functionalization. This example appears to be particularly appealing, both in terms of cost and time effectiveness, reducing the number of materials and techniques required to get to the final results, and also in terms of repeatability, limiting the error introduced by manual sample delivery. The LOD = 2.4 mM, sensitivity = 2.2 ± 0.08 µA/mM and RSD lower than 8% confirmed the effectiveness of AJP to realize a fully printed platform and of the sum of a single well in contributing to the enhancement of the overall sensitivity in a clinically relevant range (3–10 mM). Other interesting examples of fully printed electrochemical biosensors integrated into a microfluidic structure can be found in the recent literature [153], where researchers try to improve the LOD and the repeatability of point-of-care devices for biomarker detection while, at the same time, enabling a low cost and disposability [154].

## 4. Opportunities of Printed Approach for the Main Classes of Bio-Analytes

All the opportunities and metrological challenges of printed electrochemical biosensors discussed up to now need to be carefully considered, taking into consideration the specific target bio-analytes of interest: live cells, nucleic acids, proteins, metabolites and electrolytes [73,155] (Table 3). Next, we provide an overview of the most recent and relevant opportunities and trends that printed technologies are making available for each class of bio-analytes.

### 4.1. Cells and Pathogens

Process flexibility and the low cost of the fabrication of emerging printing strategies combined with the non-invasiveness of most of the electrochemical techniques make printed electrochemical sensors ideal for cell monitoring and pathogen detection [170]. When dealing with a live target, a primary issue for any inks and substrates becomes biocompatibility. Thus, an optimal interaction between cells and substrates is fundamental to ensuring effective sensing, even before electronic performances. An additional concern is related to the high humidity and salinity that printed biosensors need to undergo when inserted into a typical cell culture environment, in samples with pathogens or those implanted in a human body. Particular attention has been recently paid to improving the reliability and standardization of the outcomes of cytocompatibility tests to support researchers during the design of printed biosensors [171,172,173].

Printed electrochemical biosensing of whole cells represents a useful tool to merge the advantages of electrochemical techniques for cell monitoring with the opportunities offered by printed approaches [174]. In particular, impedance spectroscopy, due to its non-invasiveness, intrinsic label-free protocol and possibility to be applied both in 2D and 3D strategies, is one of the most widespread for these target analytes [156]. The most updated examples of cell monitoring are trying to exploit 3D printing strategies to enable the integration of sensing elements with devices capable of providing mechanical and chemical stimuli to live cells [175], and to combine imaging with electrical monitoring.

To deal with the detection of prokaryotic cells (virus, bacteria), often present in concentrations lower than fM, highly sensitive voltammetries are required. Techniques such as differential pulse or anodic stripping voltammetry are able to enhance the analyte contribution against a noisy background, reaching limits of detection in the order of fM [176,177,178], taking particular advantage of the innovative opportunity of directly printing nanostructures or biomolecules with highly controlled coatings. Other interesting recent examples also demonstrated how printed potentiometric sensors represent a reliable tool to quantify the presence of bacterial cells forming biofilms on medical surfaces, thanks to the negative correlation between the open circuit potential and the amount of bacteria [179].

### 4.2. Nucleic Acids

The possibility to develop low-cost, sensitive and rapidly printed biosensors for nucleic acid quantification is of particular interest in the field of diagnostic and screening tests, since those targets are typically key indicators of cells, viruses and bacteria, and are often responsible for pathological conditions [180]. Thus, the development of printed devices that are usable outside hospitals and laboratories is highly requested to limit pathological spread and/or to optimize clinical management, as recently strongly highlighted by the pandemic due to SARS-COV-2 [181]. However, if, on the one hand, the design of reliable and competitive printed electrochemical biosensors for those targets is particularly attractive, on the other hand, it is also very challenging due to the high standards offered by the currently adopted molecular techniques (e.g., polymerase chain reaction (PCR)). Thus, traditional techniques are affected by long processing times and the high cost of instrumentation and reagents, but they remain the current gold standard, since they are the only techniques able to reach LODs down to few copies of DNA/RNA [182]. The enormous advances in terms of nanostructure–DNA hybrid structures, ultra-high-resolution printing techniques, nano-inspired biomaterials and enhanced electrochemistry protocols accelerated the possibility of obtaining comparable sensitivity and accuracy, with lower costs and faster protocols [161,183]. Several rapid tests have been proposed in the last decade to detect most relevant viruses, such as HIV, influenza, pneumonia, all with an attempt to face the open challenges both in terms of metrology and low-cost materials to maximize test diffusion [170]. Both amperometric and impedimetric techniques have been recently proposed [184]. The most common detection strategy is based on converting a hybridization event, taking place when the target sequence recognizes its complementarity, into a quantifiable electrical signal [185,186]. Clearly, an amplification of the signal needs to be implemented in order to reach a competitive LOD. Different amplification strategies have been proposed, demonstrating the possibility to reach femto- and atto-molar concentrations of DNA [187,188], thus improving the sensitivity by almost six orders of magnitude compared to standard quantification without amplification [189]. The methods can be grouped into the following categories: (i) enzyme mediated, exploiting the recycle of a single event by the biocatalytic reaction mediated; (ii) nanomaterial-based, exploiting the high surface area of nanoparticles for the high loading of DNA probes [53]; (iii) nucleic acid-based approaches, implementing the local isothermal amplification of the DNA copies before quantification [161]. Of course, to implement most of these portable nucleic acid sensing approaches, the opportunity provided by the printing approach has been exploited, particularly in terms of the integration of the sensing elements with more complex microfluidics polymers or paper [185], [190,191]. This is essential for an accurate quantification of those targets, enabling the possibility to combine sample preparation, purification, amplification and final sensing in the same portable chip [192,193]. Furthermore, the high accuracy obtained in controlling the functionalization of nanostructures and the direct printing of small molecules or specific sequences of DNA and RNA aptamers [194] is opening the door to novel, low-cost, single-use, sensitive tests for very specific applications—for example, single mutation identification [186].

### 4.3. Proteins

Printed electrochemical biosensors provide novel opportunities for the quantification of proteins, which are peculiar, predictive, diagnostic and prognostic biomarkers of pathophysiological processes [195]. The search for novel protein biomarkers is particularly active for pathologies like cardiovascular disease, cancer or neurodegenerative diseases [196], for which the possibility to rely on novel, low-cost, ultrasensitive, accurate printed biosensors could help to take a step towards early detection, prompting intervention and drug discovery [8,197]. Thus, the early stage protein in particular might be found in blood or in other fluids in concentrations < pg/mL, which are hardly detectable with standard protein analysis [198]. Nowadays, novel printed biosensors have reached LODs of several orders of magnitude lower than the µM range of bulk electrodes, relying on the combination of highly organized novel nanostructures [69,199], magnetic immobilization [200], miniaturized geometries [198], optimized designs to enhance electrochemical parameters and flow control [201,202]. When aiming for large scale and low-cost diagnostic screening, the opportunity for the integration of printed electrochemical sensors with 3D printed modular point-of-care or lab-on-a-chip structures represent a winning strategy [203] with respect to bulky and more expensive optical and mechanical biosensors [133,204]. The techniques adopted for protein quantification using printed electrochemical sensors can mainly be categorized as label free and label based or impedance and voltammetry based. The choice of label-free or label-based techniques strongly depends upon the peculiar characteristics of the protein: molecular weight, electroactivity, surface charge, conformations, trade-off between accuracy, sensitivity and rapidity of analysis required. The impedimetric detection of proteins has been highlighted as a promising label-free tool in recent publications [205,206], combined with novel strategies such as nanostructures or molecular imprinted polymers to increase its sensitivity. Its intrinsic advantage is related to the low complexity of the protocol and the immediate correlation of the protein concentration and impedance value, without additional labels [207]. Voltammetries remain the most promising techniques in terms of the limit of detection and their customization, even if most of the detection protocols, enzyme or label mediated, are still affected by quite a high variability [208]. Alternative interesting detection principles have also been shown in [193] where a preliminary example of inkjet-printed top-gate BioFETs was used for monitoring an immunoreaction by measuring changes in the drain current, paving the way for further use of these types of devices in protein sensing.

### 4.4. Metabolites and Electrolytes

The development of low-cost, miniaturized, conformable, robust and non-invasive printed electrochemical biosensors for metabolites and electrolytes is attracting more and more interest, in particular with the recent advances in terms of wearable devices and remote sensing [135,209,210]. Thus, since metabolites and electrolytes represent relevant indicators of physio-pathological health found in multiple human fluids (e.g., blood, sweat, saliva), their accurate non-invasive continuous quantification with portable printed devices could serve as a crucial indicator for the prompt detection of a state of alarm, as interestingly highlighted by the most recent research in terms of eHealth and telemedicine [211,212]. The levels of the most common metabolites in human fluids in concentrations usually not lower than µM make them perfect candidates for electrochemical sensing, considering that the LOD of µM can be reached even without nanostructures [88]. However, in order to provide continuous monitoring, peculiar specifications need to be taken in consideration in the design of printed electrochemical biosensors for this class of target analytes: the long-term stability of the materials [213], a proper integration with microfluidic devices to continuously provide the sample to the sensing area [214] and a transduction method compatible with long-term analysis [215].

Regarding metabolites, such as glucose and lactate, the traditional chronoamperometric enzymatic detection techniques have been strongly improved in the last decade thanks to mediators, nanostructures, and a combination of different printable materials and multisensory platform implementation [216]. However, in recent years, several examples of non-enzymatic detection have been proposed [217,218], exploiting the sensitivity of peculiar printable materials and trying to point toward more efficient long-term monitoring. Regarding electrolytes (Na, K, Cl), they represent a key indicator for critical physical and mental health [219]. Their concentration, ranging from µM to mM, can be quantified both in blood and sweat by potentiometric detection due to their intrinsic charges. The state-of-the-art sensing capabilities for potassium, sodium, and pH are ≈10 µA dec^−1^ [220]. Traditionally, using non printed devices, or with commercially available Screen Printed Electrodes (SPEs), the selectivity is ensured by adopting selective membrane. The development of customized Ion Selective Electrodes (ISEs) is particularly focused on novel sensitive and selective printable materials that could substitute the membranes, and improve the electrochemical coupling between the sensing material and target analytes [221].

The active challenges for printed biosensors for metabolites and electrolytes are focused on their effective integration with wearable devices, improving the stability over long periods. In this way, the combination of additive manufacturing with proper microcontrollers and correction algorithms is bringing a real revolution, allowing for the fabrication of a whole new generation of glucose, lactate and electrolyte sensing applications, with embedded electronic and microfluidic control [222].

## 5. Conclusions

The reviewed research activities spanning across the last two decades in order to highlight how the relevance of electrochemical printed biosensors is widely recognized in fields, including basic research, regenerative medicine, in-hospital analyses and home-based point-of-care. In particular, the possibility of relying on sensitive, robust and low-cost biosensors represents a significant perspective that could create a revolution for the early diagnosis of degenerative and chronic pathologies, in the treatment and control of infectious diseases and in the development of novel solutions for tissue engineering.

Key aspects emerging from our literature analysis highlight the potential that printing technologies can bring to electrochemical biosensors in terms of miniaturization, nano-structuration, novel bio-mimetic materials and non-conventional substrates, as well as integration with microfluidics and embedded electronics. Thus, from a fabrication point of view, promising trends are represented by novel inks and non-conventional substrates for lowering the costs of biosensor fabrication (e.g., paper- or carbon-based materials) [223], by enhanced control of direct surface electrode modifications with nanostructures [98] or binding molecules for the enhancement of sensors’ sensitivity and specificity [224]. From a design point of view, promising opportunities are represented by cost-effective realization of fully printed integrated solutions [225], providing customized electronic hardware for ensuring proper biosensors conditioning and wireless data transmission [226], microfluidic circuits to ensure sample preparation, distribution and immobilization and effective strategies for continuous biosensing [214].

Exploiting these opportunities for biosensor fabrication, transduction principles and integration, printing technologies can offer a relevant potential to enlarge the field of application and to face the metrological challenges still affecting biosensing. Thus, an improved signal-to-noise ratio and LODs using nanostructure printing, the reduced cross-correlation by novel printable selective materials, and the increased repeatability and stability achieved with improved curing and printing strategies, represent leading paths that could really help biosensors to make a step towards data validation, robustness and reliability, enabling their commercialization and trusted use by medical personnel and clinical laboratories.

## Figures and Tables

**Table 1:** Main fabrication techniques for electrochemical biosensors: advantages and challenges (referenced articles are limited to the recent literature focusing on critical evaluation of positive and challenging aspects of the reported techniques).

Fabrication Techniques	Advantages	Challenges	Refs
**Bulk Electrodes**	higher stability, larger surface	no possibility of miniaturization, large volumes of sample needed, low customization possibility	[30,31]
**Printing Technologies**	miniaturization, low cost, wide range of inks and substrates available, integrability, complex geometries, possible combination with nanostructures, with bio-receptors	stability, repeatability, compatibility among materials	[4,8,25,32]
**Thin Film (Vacuum-Based, Spin Coating)**	fine control of the thickness, low costs, high repeatability	high temperatures, vacuum needed, non-compatible with low-melting point substrates, no complex geometries	[33,34,35]
**Lithography**	high resolution, high accuracy, high repeatability	long process, needed particular materials, mask based, high costs, limited available substrates	[15,36,37]
**Electrospray, Electrospinning**	good control of fibers, control of porosity, possibility to combine multiple materials	low lateral resolution, no complex geometries	[38,39,40]

**Table 2:** Review of main advantages and challenges of the three main groups of electrochemical techniques (referenced articles are limited to the recent literature focusing on critical evaluation of positive and challenging aspects of the reported techniques).

	Detectable Analyte Concentration	Advantages	Challenges	Ref
**Amperometry/Voltammetry**	lower than 10^−12^ M	highest sensitivity, high specificity, continuous monitoring, possibility to detect many compounds with different characteristic potentials in one measurement	required electroactivity, current production, interferences, effect of surrounding environment, long-term stability (degradation of materials or of labels), time-consuming	[49,61,62,63]
**Impedance spectroscopy/Conductometry**	~10^−8^ M (some recent example down to ~10^−12^ M)	miniaturization, limited invasiveness, several information frequency-dependent, direct real-time monitoring, no references electrode needed, no need for redox probe (label free)	need nanotechnologies to improve sensitivities, potential error due to double layer capacitance of non-target analytes, intrinsic non-specificity, mathematical modeling needed to extract information	[53,54,64,65,66]
**Potentiometry**	~10^−8^ M	simple conditioning, miniaturization, real-time monitoring, no current flowing, limited invasiveness, no electroactivity required	intrinsic non-specificity, very sensitive to temperature changes, possible ionic buffer interferences, frequent recalibration needed	[62,67,68,69]

**Table 3:** Review of the main advantages, challenges and trends of the main target analytes for electrochemical printed biosensors (referenced articles are limited to the recent literature focusing on critical evaluation of positive, challenging aspects and trends of each class of analyte).

	Advantages	Challenges	Main Trends	Ref
**Whole Cells (Eukaryotic and Pathogens)**	direct detection without need for sample pre-treatment to extract and purify sample, long life-time, higher stability during time	low selectivity, challenging the detection with high sensitivity, risk of contamination, often slow reactions	organic printed biosensors, degradable sensing elements, sensors integrated in glassware and scaffolds, use of disposable non-conventional substrates, use of nanostructures to enhance sensitivity	[5,93,156,157,158]
**Nucleic Acids**	wide range of application, high specificity	needed labels, time consuming because of purification step required, high costs	nanostructures, nano-hybrid materials, combine amplification techniques with the electrochemical detection	[159,160,161,162,163]
**Proteins**	simplicity, broad spectrum of applications, well-known structure, small dimensions, sensitivity, broad range of available recognition elements with high selectivity and strong binding interaction, ease validation	poor chemical, thermal and pH stability, risk of degradation due to substrate–protein interaction, high costs of antibodies for ensure selectivity, immunogenicity	low-cost disposable materials, simplify protocols, use of direct biomolecules printing, imprinted polymers, composite materials	[164,165,166]
**Metabolites and Electrolytes**	indirectly correlated with a plethora of physio-pathological processes, detectable in multiple body fluids, ideal for non-invasive continuous monitoring of health	long-term stability of enzymes, interferences of charged non-target analytes	novel selective materials, improve integration of sensors and microfluidic circuit	[167,168,169]
